# Functional hypothalamic amenorrhea and its influence on women’s health

**DOI:** 10.1007/s40618-014-0169-3

**Published:** 2014-09-09

**Authors:** B. Meczekalski, K. Katulski, A. Czyzyk, A. Podfigurna-Stopa, M. Maciejewska-Jeske

**Affiliations:** Department of Gynecological Endocrinology, Poznan University of Medical Sciences, Poznan, Poland

**Keywords:** Hypothalamic amenorrhea, Gonadotropin-releasing hormone, Bone mass density, Cardiovascular system, Sexual problems

## Abstract

**Introduction:**

Functional hypothalamic amenorrhea (FHA) is one of the most common causes of secondary amenorrhea. There are three types of FHA: weight loss-related, stress-related, and exercise-related amenorrhea. FHA results from the aberrations in pulsatile gonadotropin-releasing hormone (GnRH) secretion, which in turn causes impairment of the gonadotropins (follicle-stimulating hormone and luteinizing hormone). The final consequences are complex hormonal changes manifested by profound hypoestrogenism. Additionally, these patients present mild hypercortisolemia, low serum insulin levels, low insulin-like growth factor 1 (IGF-1) and low total triiodothyronine.

**Aim:**

The aim of this work is to review the available data concerning the effects of FHA on different aspects of women’s health.

**Results:**

Functional hypothalamic amenorrhea is related to profound impairment of reproductive functions including anovulation and infertility. Women’s health in this disorder is disturbed in several aspects including the skeletal system, cardiovascular system, and mental problems. Patients manifest a decrease in bone mass density, which is related to an increase in fracture risk. Therefore, osteopenia and osteoporosis are the main long-term complications of FHA. Cardiovascular complications include endothelial dysfunction and abnormal changes in the lipid profile. FHA patients present significantly higher depression and anxiety and also sexual problems compared to healthy subjects.

**Conclusions:**

FHA patients should be carefully diagnosed and properly managed to prevent both short- and long-term medical consequences.

## Introduction

### Definition

Functional hypothalamic amenorrhea (FHA) is classified as hypogonadotropic hypogonadism related to an aberration of the pulsatile release of gonadotropin-releasing hormone (GnRH) from the hypothalamus [1, 2]. The spectrum of hypothalamic–pituitary disturbances in FHA may be very broad and includes a lower mean frequency of LH pulses, the complete absence of LH pulsatility, as well as a normal-appearing secretion pattern and higher mean frequency of LH pulses [3]. In turn, decreased gonadotropin secretion leads to reduced estradiol production in the ovary. The disturbed hypothalamic–pituitary-ovarian axis in FHA cases is associated typically with stress, weight loss and/or excessive physical exercise and is one of the most common causes of secondary amenorrhea. Depending on the eliciting factor, there are three classes of FHA: weight loss related, stress related and exercise related [4].

Regardless of the specific trigger, a complex state of hypoestrogenism, other endocrinological aberrations and metabolic abnormalities due to FHA may impact the whole body homeostasis [5].

### Epidemiology

Secondary amenorrhea, which is defined as 3 months absence of menstruation, occurs in approximately 3–5 % of adult women. According to the American Society of Reproductive Medicine, FHA is responsible for 20–35 % of secondary amenorrhea cases and approximately 3 % of FHA cases of primary amenorrhea [6]. The incidence is higher in athlete women. DeSouza et al. [7] estimated that approximately 50 % of women who exercise regularly experience subtle menstrual disorders and approximately 30 % of women have amenorrhea. The complex of distorted eating, amenorrhea and osteoporosis was first described in 1997 and is known as female athlete triad [8].

### Differential diagnosis

Functional hypothalamic amenorrhea should be, in each case, differentiated from other forms of primary or secondary amenorrhea. The basic approach to this distinction is an assessment of the gonadotropins and identifying hypogonadotrophic hypogonadism [6]. If such a diagnosis has been made, the key diagnostic tool is a GnRH stimulation test, which in the case of FHA shows a positive response of the gonadotropins to exogenous GnRH. This test easily distinguishes hypothalamic dysfunction from pituitary diseases, where hypogonadism is also characteristic [4, 6]. Once the hypothalamic origin has been found, it is important to rule out genetic and organic diseases. As a potential cause of amenorrhea of genetic origin, the following should be taken into account: Kallman syndrome (characterized by anosmia, specific mutations), Prader–Willi syndrome (with characteristic hyperorxia, obesity, retardation) and other rare syndromes with idiopathic hypogonadotropic hypogonadism [3, 6, 9]. Features such as delayed puberty, primary amenorrhea and the presence of additional symptoms (anosmia, mental retardation, extreme obesity, facial dysmorphia, malabsorption) are suggestive of congenital diseases [6, 9]. To rule out organic diseases of the hypothalamic area (neoplasms, sarcoidosis, tuberculosis, parasitoids and other infiltrating lesions) imaging evaluation might be helpful [6].

### Hormonal disturbances associated with FHA

As described above, FHA results from depression of the hypothalamic–pituitary–ovarian axis. Impairment of GnRH and gonadotrophin secretion as a sequel is the key pathology in this trait, and also other pituitary hormone secretion is abnormal in FHA.

A typical feature of FHA is hypothalamic–pituitary–adrenal axis activation, related to stressing factors, and this phenomenon is believed to be one of the important pathogenetic factors in FHA patients [4, 10]. Increased corticotropin-releasing hormone (CRH) secretion results in an increased secretion of adrenocorticotrophin from the pituitary and cortisol from the adrenal glands, and these phenomena are linked to a reduced GnRH drive. Elevated serum and also cerebrospinal cortisol concentrations have been reported in FHA [9].

Disturbances in the hypothalamic–pituitary–thyroid axis in FHA patients are also observed. They include a low-to-normal level of thyrotropin, an increased level of reverse triiodothyronine and a low level of triiodothyronine [2].

Other findings related to hypothalamic amenorrhea include elevated nighttime serum growth hormone levels and lower 24 h prolactin levels [11]. FHA patients are characterized by low serum insulin and insulin-like growth factor 1 (IGF-1) and increased insulin sensitivity [10]. Besides this, androgen levels are known to be lower in FHA patients in comparison to healthy controls.

Clinically, FHA should not be understood only as a symptom such as amenorrhea. This disorder has a more thought-provoking clinical image [2–4]. The final endocrinological consequence of impairment in GnRH and gonadotropin pulsatile secretion is profound hypoestrogenism. Hypoestrogenic status has a negative influence on different aspects of female health, not only in menopausal women but also in young individuals [2–4]. Moreover, hormonal disturbances are accompanied by metabolic disorders related to FHA causative factors, i.e., negative energetic balance and stress [1–5].

Particularly in young women, normoestrogenism and metabolic homeostasis have a critical significance for normal bone metabolism, the cardiovascular system and mental health. Therefore, prolonged hypoestrogenism which occurs in young women may have important consequences on women’s future health.

### Neuroendocrine alterations in FHA

The precise mechanisms underlying the pathophysiology of FHA are very complex and unclear. Numerous neuropeptides, neurotransmitters and neurosteroids play an important role in the physiological regulation of GnRH pulsatile secretion and there is evidence that these substances may be involved in the pathophysiology of FHA [12]. Particular attention should be paid to such substances as kisspeptin, neuropeptide Y (NPY), ghrelin, leptin, corticotropin-releasing hormone (CRH), b-endorphin and allopregnanolone.

Kisspeptin, the product of the KiSS-1 gene and its G protein-coupled receptor GPR54, plays a master role in the puberty period and fertility [13]. The kisspeptin/GPR54 system activates the hypothalamus–pituitary–ovarian axis. Kisspeptin can directly stimulate GnRH secretion from the arcuate nucleus of the hypothalamus [14]. Administration of exogenous kisspeptin causes an increase of serum LH and FSH in healthy females. Kisspeptin administered in an acute way to women with FHA potently stimulates gonadotropin release [15].

Neuropeptide Y (NPY) acts as a regulator of energy balance, sexual behavior and circadian rhythm [16]. NPY affects the appetite center in the hypothalamus and stimulates feeding behavior [17]. NPY induces the production of GnRH if the concentrations of sex steroids, mainly estradiol, are high enough. An inhibitory effect of NPY on GnRH is observed in hypoestrogenic subjects [18]. Meczekalski et al. [19] observed lower basal serum NPY in amenorrheic patients than in menstruating women. The numbers of NPY and LH peaks were higher in patients with weight loss-related amenorrhea than in controls.

Ghrelin is a peptide that stimulates appetite, but reduces fat utilization [20]. Additionally, ghrelin inhibits the hypothalamic–pituitary–gonadal axis and is responsible for the prolongation of amenorrhea in subjects who have regained normal weight [21]. Women with FHA are characterized by elevated ghrelin levels compared with healthy women [22]. Exercising or underweight amenorrheic patients are characterized by a significantly greater serum ghrelin elevation than those who remain with stable weight [23].

Leptin is an adipose tissue-derived hormone which plays a crucial role in the link between metabolic and hormonal signals and their impact on the reproductive axis [24]. Patients suffering from hypothalamic amenorrhea are characterized by considerably lower serum leptin concentrations compared with age-, weight- and body fat-matched eumenorrheic controls [25].

CRH plays an important role in the regulation of reproduction by modulating the hypothalamus–pituitary–adrenal axis as well as the hypothalamus–pituitary–ovary axis [26]. Physical or mental stress activates an instant increase of CRH in the CNS. Subsequently, CRH stimulates the pituitary secretion of ACTH and other pro-opiomelanocortin (POMC)-related peptides such as beta-endorphin and b-lipotropic hormone. Increased secretion of glucocorticosteroids inhibits the release of GnRH and gonadotropins. These described mechanisms suggest a specific stress etiology of hypothalamic amenorrhea [27].

Beta-Endorphin is an endogenous neuropeptide, found in the hypothalamus and pituitary gland, playing a significant role in the etiopathophysiology of hypothalamic amenorrhea [28]. This effect is based on the disturbed GnRH production and thus impaired LH release. CRH may directly inhibit GnRH secretion and stimulate beta-endorphin production in the case of stress-related and exercise-related amenorrhea. These two conditions are characterized by increased opioidergic activity in stress- and exercise-related amenorrhea [27].

Allopregnanolone is a neurosteroid acting as an endogenous modulator of excitability of the CNS [29]. Patients suffering from hypothalamic amenorrhea demonstrate a considerable episodic release of allopregnanolone. This specific pulsatile secretion is similar in frequency and amplitude to that in fertile women. However, a higher pulse amplitude of allopregnanolone is demonstrated in amenorrheic patients than in controls. Likewise, higher plasma allopregnanolone levels are observed in amenorrheic subjects [30].

## Reproduction

Functional hypothalamic disturbances and neuroendocrine aberrations have both short- and long-term consequences for reproductive health. Understandably, an impaired or diminished hypothalamic-pituitary–ovarian axis leads to anovulation and hypoestrogenism. Lack of cyclical changes of estradiol and progesterone concentrations leads to abolished endometrial cyclicity and typically endometrium is in the persistent early profilerative phase. Anovulation is directly linked to the neurohormonal and hormonal background of FHA. Impairment of pulsatile GnRH secretion causes the impairment of pulsatile LH and FSH secretion. The final consequence is profound hypoestrogenism which determines anovulation [31]. If the disturbance appears during puberty, women present with primary amenorrhea. Secondary amenorrhea, which is more common in FHA, develops in postpubertal girls and women.

According to Hind [31], proper diagnosis and treatment of FHA are important due to the potential risk of infertility arising from chronic amenorrhea. Devoto and Aravena [32] found that in teenagers with hypothalamic dysfunction and menstrual disturbances, a deficient or bad response to clomiphene does not necessarily indicate a bad prognosis in terms of menses or fertility.

As stated before, anovulation is a characteristic feature of FHA, so those patients who suffer from this condition are unable to become spontaneously pregnant—a fact that is well established [33]. Another issue is how long lasting untreated hypothalamic amenorrhea influences reproductive health in the future. Delayed menarche, dyschronic puberty and the underdevelopment of secondary and tertiary sex characteristics are potential obstacles to reproductive health in girls affected by FHA during puberty. In adult women, the same disease can lead to atrophic changes in the urogenital mucosa and in the muscles of the uterus.

However, if a patient with FHA or with a history of FHA becomes pregnant, this pregnancy requires special care due to the increased risk of miscarriage and preterm labor [34]. Additionally, patients with FHA can present more obstetric complications such as impaired weight gain and compromised intrauterine fetal growth [35].

Shen ZQ et al. [36] studied factors affecting menstrual resumption and estimated the pituitary response to GnRH in functional hypothalamic amenorrhea. They recruited to the study 30 cases with FHA and treated them with continuous 1 mg/day estradiol valerate orally. The occurrence of at least three consecutive regular cycles was seen in the majority of FHA women undertaking estrogen replacement therapy. The likelihood of recovery was affected by their BMI beforehand and by amenorrhea, but not by weight gain during therapy. The menstrual resumption in FHA was accompanied by the recovery of serum LH and the LH response to GnRH [32].

## Bone health

FHA exerts a negative influence on the skeletal system. It is related to a great extent to the failure to achieve peak bone mass (PBM) [33]. PBM is defined as the largest amount of bone tissue that a person has at any point in life [37]. Most people reach their peak mass by the age of 30 years, but approximately 40–50 % of PBM is formed during the puberty period [33]. Hormonal and nutritional factors contribute 40–60 % and genetic factors 60–80 % in constituting PBM [38]. Sex steroids (estrogens, androgens), growth hormone (GH) and insulin growth-like 1 (IGF1) are the main hormones which exert a positive influence on PBM formation [39]. In young women, estrogens are the critical determinants which ensure proper bone metabolism [40]. Estrogen action is performed in three directions: the activation of bone remodeling units, the suppression of bone resorption and the stimulation of bone formation [41]. Estrogens stimulate synthesis of the main growth factors such as transforming growth factor beta-TGF-beta, bone morphogenetic protein 6-BMP6 and insulin-like growth factor 1-IGF-1. Estrogens are also responsible for 1,25(OH)D3 expression receptors increase [42]. Additionally, estrogen can exert an influence on the inhibition of RANKL production (the receptor activator of nuclear factor kappa B ligand) and an increase of osteoprotegerin gene expression (the inhibitor of osteoclasts formation). Moreover, estrogens are responsible for the decrease of proresorptive cytokins’ synthesis such as macrophage-colony stimulating factor (M-CSF), interleukin-6 (IL-6), interleukin-1, (IL-1) and tumor necrosis factor α (TNF-α) [43]. Therefore, prolonged hypoestrogenism in young women with FHA is associated with osteopenia and osteoporosis risk.

The minimal serum estradiol level, which has a positive impact on bone metabolism, is 40–50 pg/ml [44]. According to the literature and clinical practice, serum estradiol levels in patients with FHA are below 20 pg/ml [36]. Patients with FHA are threatened by a low PBM not only due to hypoestrogenism. Other important factors include improper diet (low calcium and vitamin D3 intake), undernutrition and also excessive exercises [45]. Excessive exercise (exercise-related hypothalamic amenorrhea) does not improve bone mass density (BMD), but leads to osteopenia [46]. Hormonal factors responsible for a decrease of bone mass density are as follows: low serum IGF-I, insulin and high serum cortisol [42]. Generally, the degree of BMD decrease in FHA patients is less expressed than in patients with anorexia nervosa [47, 48].

It is important to stress that the female sex per se is more prone to have osteopenia or osteoporosis because the optimal PBM reached by females is 25–30 % lower than in males [49]. Reduction of PBM increases the risk of a decreased bone mass density during the pubertal period, as well as in the fertile and perimenopausal periods, which results in a major risk of pathological fractures [40].

Podfigurna-Stopa et al. [50] have estimated the skeletal status and body composition of young women with functional hypothalamic amenorrhea. FHA individuals had a decreased fat tissue mass and an imbalanced relationship between body weight, fat tissue mass and lean body mass. Despite the reduced BMD and bone mineral content, FHA did not, however, significantly affect bone strength.

According to the International Society for Clinical Densitometry, amenorrhea related to hypoetrogenism lasting 6 months is the indication to perform a densitometry (DEXA) of the spinal column [51]. This has an important clinical aspect because FHA as a disorder of the hypothalamic–pituitary axis is the main entity responsible for BMD decrease in young women [52].

More attention should be paid to exercise among women. For most of the young women, exercise causes a positive effect—improving health and physical fitness. However, if the exercise energy expenditure increases more than energy intake, a variety of clinical manifestations can occur. Such sports as ballet dancing, running, or gymnastics may lead to poor nutritional behaviors, resulting in low energy availability. Abnormally low BMD and osteoporosis in exercising women relate to premature bone loss and microarchitectural deterioration. Mainly, female athletes with amenorrhea are at increased risk for stress fractures and skeletal fragility [53].

## Cardiovascular system

Cardiovascular disease (CVD) is the leading cause of death in women in developed countries and, interestingly, proportionally more women die from CVD than men.

As it is known, hypoestrogenism can interfere with the cardiovascular system function in many ways. Coronary and peripheral vessels contain estrogen receptors that permit estradiol to play a regulatory role in vascular function. Estrogen excites the synthesis of nitric oxide (NO) through both genomic and nongenomic effects, leading to the augmented production of endothelial-derived NO, causing vasodilatation [54].

Estradiol exerts a positive, cardioprotective effect through its influence on the endothelial, myocardial and vascular function and metabolic parameters [55]. In contrast, hypoestrogenism can lead to endothelial dysfunction, an impaired bioactivity of nitric oxide, perturbation in autonomic function, activation of the rennin–angiotensin system and lipid profile changes [56].

These physiological and pathological phenomena are reflected in clinical studies. Several investigators have demonstrated a correlation between FHA and endothelial dysfunction [57]. It was clearly shown that the flow-mediated dilation of the brachial artery (which is a precise predictor of coronary endothelial dysfunction) is impaired in women with FHA [51]. It is suggested that the decrease of endothelial NO bioavailability is caused by chronic estrogen deficiency. Moreover, some authors have proved the protective effect of exogenous estrogens in young women against impaired vessels’ dilatation. Rickenlund et al. [58] documented significantly increased brachial artery dilatation after 9 months of treatment with low-dose combined contraceptives (30 µg of ethinyl estradiol and 150 µg of levonorgestrel): from 1.42 ± 0.98 % before treatment to 4.88 ± 2.20 % during treatment.

A Women’s Ischemia Syndrome Evaluation (WISE) study found a significant association between premenopausal angiographic coronary artery disease (CAD) and hypothalamic hypogonadism [59]. O’Donnell et al. [60] recently showed that young athletes with chronic hypoestrogenemia displayed an impaired peripheral vascular function that was combined with lower resting blood pressures and heart rate and reduced ischemic responses to occlusion challenge compared to ovulatory women.

Impaired cardiovascular function in hypothalamic amenorrhea is believed to be linked mainly to hypoestrogenism, but it is also aggravated by negative energy balance and metabolic disturbances. Patients with FHA are characterized by an impaired lipid profile and are at risk of glucose metabolism abnormalities. Women with exercise-related amenorrhea present higher serum total cholesterol, LDL cholesterol, apolipoprotein B and triglyceride concentrations than healthy individuals [61, 62]. On the other hand, premenopausal women with hypoestroegism of hypothalamic origin present an increased frequency of diabetes mellitus. Moreover, Ahmed et al. [63] showed that coronary artery disease (detected in coronarography) has an increased prevalence and extent among women with diabetes and hypothalamic hypoestrogenism in comparison to women with diabetes alone.

These observations substantiate the importance of cyclic ovarian function as an indicator of cardiovascular health. However, the influence of hypoestrogenism in young women of hypothalamic origin on cardiovascular health requires further studies. Especially the issue of the long-term consequences of FHA on CVD risk needs to be cleared to possibly minimize the risk of cardiovascular events in this group of women.

## Mental and sexual dysfunction

Mood in women in its essential part is linked to serum sex steroid levels, particularly estrogen [64]. Hypoestrogenism in young women with FHA is strongly related to changes in different neuropeptides, neurotransmitters and neurosteroids activity at the brain level. Specifically, serotonin, dopamine and allopregnanolone fluctuations can modulate mood in amenorrheic women [65]. The number of studies concerning this subject is very limited. FHA patients present a specific psychological profile.

Gilles and Berga [66] assessed the association of cognitive function and emotional and psychiatric history in women with functional hypothalamic amenorrhea compared with amenorrheic and eumenorrheic controls. Women with FHA endorsed more dysfunctional attitudes, had greater difficulty in coping with daily stresses and tended to endorse greater interpersonal dependence than eumenorrheic women.

Hypercortisolemia is one of the characteristic hormonal features of FHA patients. Serum cortisol levels positively correlate with the Hamilton Rating Scale for Depression and Anxiety, and hypercortisolemia can be regarded as a possible mediator of mood disturbances [67]. FHA patients present a particular susceptibility to common life events, restrictive disordered eating, depressive traits and psychosomatic disorders [68].

Bomba et al. [69] reported that patients with FHA and AN present common psychopatological aspects. These include maturity issues, social insecurity and introversion, a tendency to depression, excessive concerns with dieting and the fear of gaining weight.

Sexual functioning is also of important significance for FHA patients. Dundon et al. [70] found that women with FHA have more sexual problems than healthy controls. Theoretically, this functioning is determined by the psychological and hormonal background. Psychological problems are aggravated by the fact that FHA is associated with anxiety, depressive symptoms and high rates of mood disorders [71]. The mediating effects of anxiety and depression may explain the occurrence of sexual dysfunction which is potentially associated with FHA. Although they were significantly higher in FHA subjects, out of the two, only depression offered a plausible explanation for sexual difficulties FHA subjects typically face. Due to the objective difficulties of correlating hormonal measurements with psychometric data, it remains uncertain whether such an association can result from a common neuroendocrine background. Furthermore, it cannot be stated that anxiety (and not depression) influences sexual function differently in FHA subjects [72].

The hormonal background of sexual dysfunction in FHA may be related to profound hypoestrogenism and hypoandrogenemia [72, 73]. It can be related to genital tract status and sexual drive.

## Conclusions

FHA is an underestimated clinical problem. It is related to profound impairment of reproductive functions including anovulation and infertility. Women’s health in this disorder is disturbed in several aspects including their skeletal system, cardiovascular system and mental problems. Patients manifest a decrease of bone mass density, which is related to an increase of fracture risk. Therefore, osteopenia and osteoporosis are the main long-term complications of FHA. Cardiovascular complications include endothelial dysfunction and abnormal changes in the lipid profile. FHA patients present significantly higher depression and anxiety and also sexual problems compared to healthy subjects.

FHA patients should be carefully diagnosed and properly managed to prevent both short- and particularly long-term medical consequences.

## References

[1] Berga SL, Mortola JF, Girton L, Suh B, Laughlin G, Pham P, Yen SS (1989). Neuroendocrine aberrations in women with functional hypothalamic amenorrhea. J Clin Endocrinol Metab.

[2] Gordon MC (2010). Functional hypothalamic amenorhea. N Engl J Med.

[3] Genazzani AD (2005). Neuroendocrine aspects of amenorrhea related to stress. Pediatr Endocrinol Rev.

[4] Meczekalski B, Podfigurna-Stopa A, Warenik-Szymankiewicz A, Genazzani AR (2008). Functional hypothalamic amenorrhea: current view on neuroendocrine aberrations. Gynecol Endocrinol.

[5] Harlow SD, Goldman MB, Katch M (2000). Menstruation and menstrual disorders : the epidemiology of menstruation and menstrual dysfunction. Women and Health.

[6] Practice Committee of the American Society for Reproductive Medicine (2006). Current evaluation of amenorrhea. Fertil Steril.

[7] De Souza MJ (2009). High prevalence of subtle and severe menstrual disturbances in exercising women: confirmation using daily hormone measures. Hum Reprod.

[8] Otis CL (1997). American college of sports medicine position stand. The female athlete triad. Med Sci Sports Exerc.

[9] Valdes-Socin H, Rubio Almanza M, Tomé Fernández-Ladreda M, Debray FG, Bours V, Beckers A (2014). Reproduction, smell, and neurodevelopmental disorders: genetic defects in different hypogonadotropic hypogonadal syndromes. Front Endocrinol (Lausanne).

[10] Berga SL, Daniels TL, Giles DE (1997). Women with functional hypothalamic amenorrhea but not other forms of anovulation display amplified cortisol concentrations. Fertil Steril.

[11] Laughlin GA, Dominguez CE, Yen SS (1998). Nutritional and endocrine-metabolic aberrations in women with functional hypothalamic amenorrhea. J Clin Endocrinol Metab.

[12] Marshall LA (1994). Clinical evaluation of amenorrhea in active and athletic women. Clin Sports Med.

[13] Funes S, Hedrick JA, Vassileva G, Markowitz L, Abbondanzo S, Golovko A, Yang S, Monsma FJ, Gustafson EL (2003). The KiSS-1 receptor GPR54 is essential for the development of the murine reproductive system. Biochem Biophys Res Commun.

[14] Roa J, Tena-Sempere M (2007). KiSS-1 system and reproduction: comparative aspects and roles in the control of female gonadotropic axis in mammals. Gen Comp Endocrinol.

[15] Sills ES, Walsh AP (2008). The GPR54-Kisspeptin complex in reproductive biology: neuroendocrine significance and im- plications for ovulation induction and contraception. Neuro Endocrinol Lett.

[16] El Bahh B, Balosso S, Hamilton T, Herzog H, Beck-Sickinger AG, Sperk G, Gehlert DR, Vezzani A, Colmers WF (2005). The anti-epileptic actions of neuropeptide Y in the hippocampus are mediated by Y and not Y receptors. Eur J Neurosci.

[17] Pedrazzini T, Pralong F, Grouzmann E (2003). Neuropeptide Y: the universal soldier. Cell Mol Life Sci.

[18] Kalra SP, Crowley WR (1992). Neuropeptide Y: a novel neuroendo- crine peptide in the control of pituitary hormone secretion, and its relation to luteinizing hormone. Front Neuroendocrinol.

[19] Meczekalski B, Genazzani AR, Genazzani AD, Warenik-Szymankiewicz A, Luisi M (2006). Clinical evaluation of patients with weight loss-related amenorrhea: neuropeptide Y and luteinizing hormone pulsatility. Gynecol Endocrinol.

[20] Barreiro ML, Tena-Sempere M (2004). Ghrelin and reproduction: a novel signal linking energy status and fertility?. Mol Cell Endocrinol.

[21] Schneider LF, Warren MP (2006). Functional hypothalamic ame- norrhea is associated with elevated ghrelin and disordered eating. Fertil Steril.

[22] Tolle V, Kadem M, Bluet-Pajot MT, Frere D, Foulon C, Bossu C, Dardennes R, Mounier C, Zizzari P, Lang F (2003). Balance in ghrelin and leptin plasma levels in anorexia nervosa patients and constitutionally thin women. J Clin Endocrinol Metab.

[23] De Souza MJ, Leidy HJ, O’Donnell E, Lasley B, Williams NI (2004). Fasting ghrelin levels in physically active women: relationship with menstrual disturbances and metabolic hormones. J Clin Endocrinol Metab.

[24] Considine RV, Caro JF (1996). Leptin: genes, concepts and clinical perspective. Horm Res.

[25] Andrico S, Gambera A, Specchia C, Pellegrini C, Falsetti L, Sartori E (2002). Leptin in functional hypothalamic amenorrhoea. Hum Reprod.

[26] Smith R, Nicholson RC (2007). Corticotrophin releasing hormone and the timing of birth. Front Biosci.

[27] Rivier C, Brownstein M, Spiess J, Rivier J, Vale W (1982). In vivo corticotropin-releasing factor-induced secretion of adrenocor- ticotropin, b-endorphin, and corticosterone. Endocrinology.

[28] Remorgida V, Venturini PL, Anserini P, Salerno E, De Cecco L (1990). Naltrexone in functional hypothalamic amenorrhea and in the normal luteal phase. Obstet Gynecol.

[29] Mellon SH, Deschepper CF (1993). Neurosteroid biosynthesis: genes for adrenal steroidogenic enzymes are expressed in the brain. Brain Res.

[30] Genazzani AD, Luisi M, Malavasi B, Strucchi C, Luisi S, Casarosa E, Bernardi F, Genazzani AR, Petraglia F (2002). Pulsatile secretory characteristics of allopregnanolone, a neuroactive steroid, during the menstrual cycle and in amenorrheic subjects. Eur J Endocrinol.

[31] Hind K (2008). Recovery of bone mineral density and fertility in a former amenorrheic athlete. J Sports Sci Med.

[32] Devoro E, Aravena L (2002). Favorable reproductive and menstrual evolution in adult women, who presented in the adolescence, menstrual disturbances by hypothalamic dysfunction and lack of response to clomiphene. Rev Med Chil.

[33] Juul A, Hagen CP, Aksglaede L, Sørensen K, Mouritsen A, Frederiksen H, Main KM, Mogensen SS, Pedersen AT (2012). Endocrine evaluation of reproductive function in girls during infancy, childhood and adolescence. Endocr Dev.

[34] EasterA TreasureJ, Micali N (2011). Fertility and prenatal attitudes towards pregnancy in women with eating disorders: results from the avon longitudinal study of parents and children. BJOG.

[35] BeckerAE GrinspoonSK, KlibanskiA HerzogDB (1999). Eating disorders. NEJM.

[36] Shen ZQ, Xu JJ, Lin JF (2013). Resumption of menstruation and pituitary response to gonadotropin-releasing hormone infunctional hypothalamic amenorrhea subjects undertaking estrogen replacement therapy. J Endocrinol Invest.

[37] Clarke BL, Khosla S (2010). Female reproductive system and bones. Arch Biochem Biophys.

[38] Gordon MC (2010). Functional hypothalamic amenorhea. N Engl J Med.

[39] Seeman E (2002). Pathogenesis of bone fragility in women and men. Lancet.

[40] Meczekalski B, Podfigurna-Stopa A, Genazzani AR (2010). Hypoestrogenism in young women and its influence on bone mass density. Gynecol Endocrinol.

[41] Khosla S, Oursler MJ, Monroe DG (2012). Estrogen and the skeleton. Trends Endocrinol Metab.

[42] Taes Y, Lapauw B, Vandewalle S, Zmierczak H, Goemaere S, Vanderschueren D, Kaufman JM, T’Sjoen G (2009). Estrogen-specific action on bone geometry and volumetric bone density: longitudinal observations in an adult with complete androgen insensitivity. Bone.

[43] Mircea CN, Lujan ME, Pierson RA (2007). Metabolic fuel and clinical implications for female reproduction. J Obstet Gynaecol Can.

[44] Crandall CJ, Tseng CH, Karlamangla AS, Finkelstein JS, Randolph JF, Thurston RC, Huang MH, Zheng H, Greendale GA (2013). Serum sex steroid levels and longitudinal changes in bone density in relation to the final menstrual period. J Clin Endocrinol Metab.

[45] De Souza MJ, Williams NI (2004). Physiological aspects and clinical sequelae of energy deficiency and hypoestrogenism in exercising women. Hum Reprod Update.

[46] Warren MP, Brooks-Gunn J, Fox RP, Holderness CC, Hyle EP, Hamilton WG (2002). Osteopenia in exercise-associated amenorrhea using ballet dancers as a model: a longitudinal study. J Clin Endocrinol Metab.

[47] Lawson EA, Donoho D, Miller KK, Misra M, Meenaghan E, Lydecker J, Wexler T, Herzog DB, Klibanski A (2009). Hypercortisolemia is associated with severity of bone loss and depression in hypothalamic amenorrhea and anorexia nervosa. J Clin Endocrinol Metab.

[48] Guo LJ (2013). Relationship between serum omentin-1 level and bone mineral density in girls with anorexia nervosa. J Endocrinol Invest.

[49] Davies JH, Evans BAJ, Gregory JW (2005). Bone acquisition in healthy children. Arch Dis Child.

[50] Podfigurna-Stopa A, Pludowski P, Jaworski M, Lorenc R, Genazzani AR, Meczekalski B (2012). Skeletal status and body composition in young women with functional hypothalamic amenorrhea. Gynecol Endocrinol.

[51] Gordon CM, Bachrach LK, Carpenter TO, Crabtree N, El-Hajj Fuleihan G, Kutilek S, Lorenc RS, Tosi LL, Ward KA, Ward LM, Kalkwarf HJ (2008). Dual energy X-ray absorptiometry interpretation and reporting in children and adolescents: the 2007 ISCD pediatric official positions. J Clin Densitom.

[52] Misra M (2008). What is the best strategy to combat low bone mineral density in functional hypothalamic amenorrhea?. Nat Clin Pract Endocrinol Metab.

[53] Lambrinoudaki I, Papadimitriou D (2010). Pathophysiology of bone loss in the female athlete. Ann N Y Acad Sci.

[54] Mendelsohn ME (2002). Protective effects of estrogen on the cardiovascular system. Am J Cardiol.

[55] Reckelhoff JF (2005). Sex steroids, cardiovascular disease, and hypertension: unanswered questions and some speculations. Hypertension.

[56] Ouyang P, Michos ED, Karas RH (2006). Hormone replacement theray and the cardiovascular system lessons learned and unanswered questions. J Am Coll Cardiol.

[57] O’Donnell E, Goodman JM, Harvey PJ (2011). Clinical review: cardiovascular consequences of ovarian disruption: a focus on functional hypothalamic amenorrhea in physically active women. J Clin Endocrinol Metab.

[58] Rickenlund A, Eriksson MJ, Schenck-Gustafsson K, Hirschberg AL (2005). Oral contraceptives improve endothelial function in amenorrheic athletes. J Clin Endocrinol Metab.

[59] Bairey Merz CN, Johnson BD, Sharaf BL, Bittner V, Berga SL, Braunstein GD, Hodgson TK, Matthews KA, Pepine CJ, Reis SE, Reichek N, Rogers WJ, Pohost GM, Kelsey SF, Sopko G (2003). Hypoestrogenemia of hypothalamic origin and coronary artery disease in premenopausal women: a report from the NHLBI sponsored WISE study. J Am Coll Cardiol.

[60] O’Donnell E, Harvey PJ, Goodman JM, De Souza MJ (2007). Long-term estrogen deficiency lowers regional blood flow, resting systolic blood pressure, and heart rate in exercising premenopausal women. Am J Physiol Endocrinol Metab.

[61] Rickenlund A, Eriksson MJ, Schenck-Gustafsson K, Hirschberg AL (2005). Amenorrhea in female athletes is associated with endothelial dysfunction and unfavorable lipid profile. J Clin Endocrinol Metab.

[62] Friday KE, Drinkwater BL, Bruemmer B, Chesnut C, Chait A (1993). Elevated plasma low-density lipoprotein and high-density lipoprotein cholesterol levels in amenorrheic athletes: effects of endogenous hormone status and nutrient intake. J Clin Endocrinol Metab.

[63] Ahmed B, Bairey Merz CN, Johnson BD, Bittner V, Berga SL, Braunstein GD, Hodgson TK, Smith K, Shaw L, Kelsey SF, Sopko G, WISE Study Group, WISE Study Group (2008). Diabetes mellitus, hypothalamic hypoestrogenemia, and coronary artery disease in premenopausal women (from the National Heart, Lung, and Blood Institute sponsored WISE study). Am J Cardiol.

[64] McEwen BS, Akama KT, Spencer-Segal JL, Milner TA, Waters EM (2012). Estrogen effects on the brain: actions beyond the hypothalamus via novel mechanisms. Behav Neurosci.

[65] Kormos V, Gaszner B (2013). Role of neuropeptides in anxiety, stress, and depression: from animals to humans. Neuropeptides.

[66] Giles DE, Berga SL (1993). Cognitive and psychiatric correlates of functional hypothalamic amenorrhea: a controlled comparison. Fertil Steril.

[67] Lawson EA, Donoho D, Miller KK, Misra M, Meenaghan E, Lydecker J, Wexler T, Herzog DB, Klibanski A (2009). Hypercortisolemia is associated with severity of bone loss and depression in hypothalamic amenorrhea and anorexia nervosa. J Clin Endocrinol Metab.

[68] Bomba M, Gambera A, Bonini L, Peroni M, Neri F, Scagliola P, Nacinovich R (2007). Endocrine profiles and neuropsychologic correlates of functional hypothalamic amenorrhea in adolescents. Fertil Steril.

[69] Bomba M, Corbetta F, Bonini L, Gambera A, Tremolizzo L, Neri F, Nacinovich R (2013). Psychopathological traits of adolescents with functional hypothalamic amenorrhea: a comparison with anorexia nervosa. Eat Weight Disord.

[70] Dundon CM, Rellini AH, Tonani S, Santamaria V, Nappi R (2010). Mood disorders and sexual functioning in women with functional hypothalamic amenorrhea. Fertil Steril.

[71] Berga SL, Loucks TL (2006). Use of cognitive behavior therapy for functional hypothalamic amenorrhea. Ann N Y Acad Sci.

[72] Marcus MD, Loucks TL, Berga SL (2001). Psychological correlates of functional hypothalamic amenorrhea. Fertil Steril.

[73] Miller KK, Lawson EA, Mathur V, Wexler TL, Meenaghan E, Misra M, Herzog DB, Klibanski A (2007). Androgens in women with anorexia nervosa and normal-weight women with hypothalamic amenorrhea. J Clin Endocrinol Metab.

